# Missed Opportunities: The Importance of HIV Screening in Adolescent Populations

**DOI:** 10.7759/cureus.113685

**Published:** 2026-07-30

**Authors:** Elizabeth C Bolton, Eric McGrath, Laura J Benajmins

**Affiliations:** 1 Department of Pediatrics, Wayne State University School of Medicine, Detroit, USA

**Keywords:** acute hiv infection, adolescent hiv screening, fourth-generation hiv testing, missed diagnosis, universal hiv testing

## Abstract

Adolescents and young adults represent a substantial proportion of individuals newly diagnosed with HIV in the United States. Despite this, many youth living with HIV remain unaware of their status. HIV screening should be discussed and offered to adolescents; however, teens frequently report not being offered testing, and healthcare providers do not consistently adhere to screening practices. Detecting early HIV infection, including acute infection, is critical to initiating treatment, preventing transmission, and understanding patterns of disease. Acute HIV detection in adolescents and young adults is particularly challenging because early clinical manifestations are often nonspecific and may be misattributed to other common illnesses, such as influenza or mononucleosis. Maintaining a low threshold for testing, a high index of suspicion for HIV even in the presence of other diagnoses, and advocating for broad screening practices are essential to preventing missed diagnoses in this population.

A 19-year-old male with a history of multiple sexually transmitted infections (STIs) was prescribed HIV pre-exposure prophylaxis (PrEP) but never initiated therapy. One year later, with a one-month prior negative fourth-generation HIV test, the patient developed a 103°F fever, back pain, myalgias, and a headache. Over the next several days, he presented to three emergency departments, but HIV testing was not performed. Subsequently, the patient presented to the clinic, and exam findings were consistent with streptococcal pharyngitis. Rapid strep testing was positive, and he was treated with intramuscular (IM) penicillin. However, serum testing from that visit showed HIV RNA PCR of 3,652,943 copies/mL (reference: undetectable, <20 copies/mL). The patient returned to the clinic two days later and was started on antiretroviral therapy.

This case highlights the critical need for routine and timely HIV screening, specifically in the adolescent and young adult population, as acute HIV infection can present with non-specific symptoms as well as concurrent diagnoses such as strep throat. Clinicians must maintain a high index of suspicion and be knowledgeable about HIV testing modalities, particularly the fourth-generation antigen/antibody tests. Universal screening should not be based on self-reported risk behaviors, as they can be undisclosed. Barriers, including stigma, confidentiality concerns, and provider discomfort, hinder testing in primary care settings.

Maintaining a high index of suspicion for HIV in adolescent and young adult patients is crucial, as missed or delayed diagnoses can accelerate disease progression and increase transmission. Integrating HIV testing into routine adolescent care helps normalize screening and reduce stigma.

## Introduction

According to the Centers for Disease Control and Prevention (CDC), youth aged 13 to 24 account for more than one in five new HIV diagnoses in the United States. In 2014, approximately 60% of youth living with HIV were unaware of their status [1]. Although HIV incidence among young people has declined in recent years, adolescents and young adults remain disproportionately affected by HIV in the United States. CDC estimates that annual new HIV infections among persons aged 13-24 years decreased by 34% between 2017 and 2021; however, substantial racial and ethnic disparities persist [2]. Organizations, such as the CDC, United States Preventive Services Task Force (USPSTF), and American Academy of Pediatrics (AAP), advocate for routine HIV screening in adolescents and young adults. The CDC recommends that HIV screening be discussed and offered at least once to individuals aged 13-64 years in health care settings, with more frequent testing for those at increased risk. Despite these recommendations, teens frequently report not being offered testing, and studies indicate that healthcare providers do not consistently adhere to screening guidelines [3].

Detecting early HIV infections, including acute infection, is critical to initiating treatment, preventing HIV transmission, and tracking HIV incidence. Early use of antiretroviral therapy has been shown to keep viral load below the point of detection through reduced replication in as quickly as three months [4]. Further, early antiretroviral therapy reduces the emergence of resistant phenotypes [5]. Unfortunately, acute HIV detection, especially in adolescents and young adults, is complicated by the fact that early HIV clinical manifestations are often misdiagnosed as other illnesses, such as influenza, mononucleosis, streptococcal pharyngitis, viral hepatitis, toxoplasmosis, or secondary syphilis. Maintaining a low threshold for testing, high suspicion for HIV even in the presence of other illnesses, and advocating for universal screening are critical to preventing missed diagnoses in this population. This case study will narrate the importance of early recognition, universal screening, and prompt treatment initiation among adolescents and young adults with HIV.

## Case presentation

A 19-year-old male with a history of multiple sexually transmitted infections (STIs) (pharyngeal gonorrhea, urine and rectal chlamydia) and over 20 lifetime partners (male and female) was prescribed Emtricitabine/Tenofovir for HIV pre-exposure prophylaxis (PrEP). Unfortunately, he never initiated therapy. One year later, in October, he tested positive for chlamydia in his urine, with all other sites negative. He denied sexual activity in the preceding two months. At this visit, a fourth-generation HIV test for detecting both HIV antibodies and the P24 antigen was negative. One month later, in November, the patient developed a 103°F fever, back pain, myalgias, and a headache. Further, he reported abdominal cramping, diarrhea, vomiting, and a sore throat. Over the next several days, he presented to three emergency departments where various workups were performed. Physical exam findings were nonspecific. Notably, HIV testing was not performed. Six days after symptom onset, the patient presented to the clinic. At this time, exam findings were consistent with streptococcal pharyngitis. Rapid strep testing was positive, and he was treated with IM penicillin. Serum testing revealed a reactive fourth-generation HIV test (reference: nonreactive), an indeterminate HIV-1 antibody result (reference: negative), an HIV-1 RNA PCR level of 3,652,943 copies/mL (reference: undetectable, <20 copies/mL), and a CD4 count of 280 cells/mm³ (reference: 500-1,500 cells/mm³).

Two days following serum testing, the patient returned to the clinic and was started on multi-drug HIV therapy of Bictegravir/Emtricitabin/Tenofovir alafenamide. 

## Discussion

Testing advocacy 

**Figure 1:**
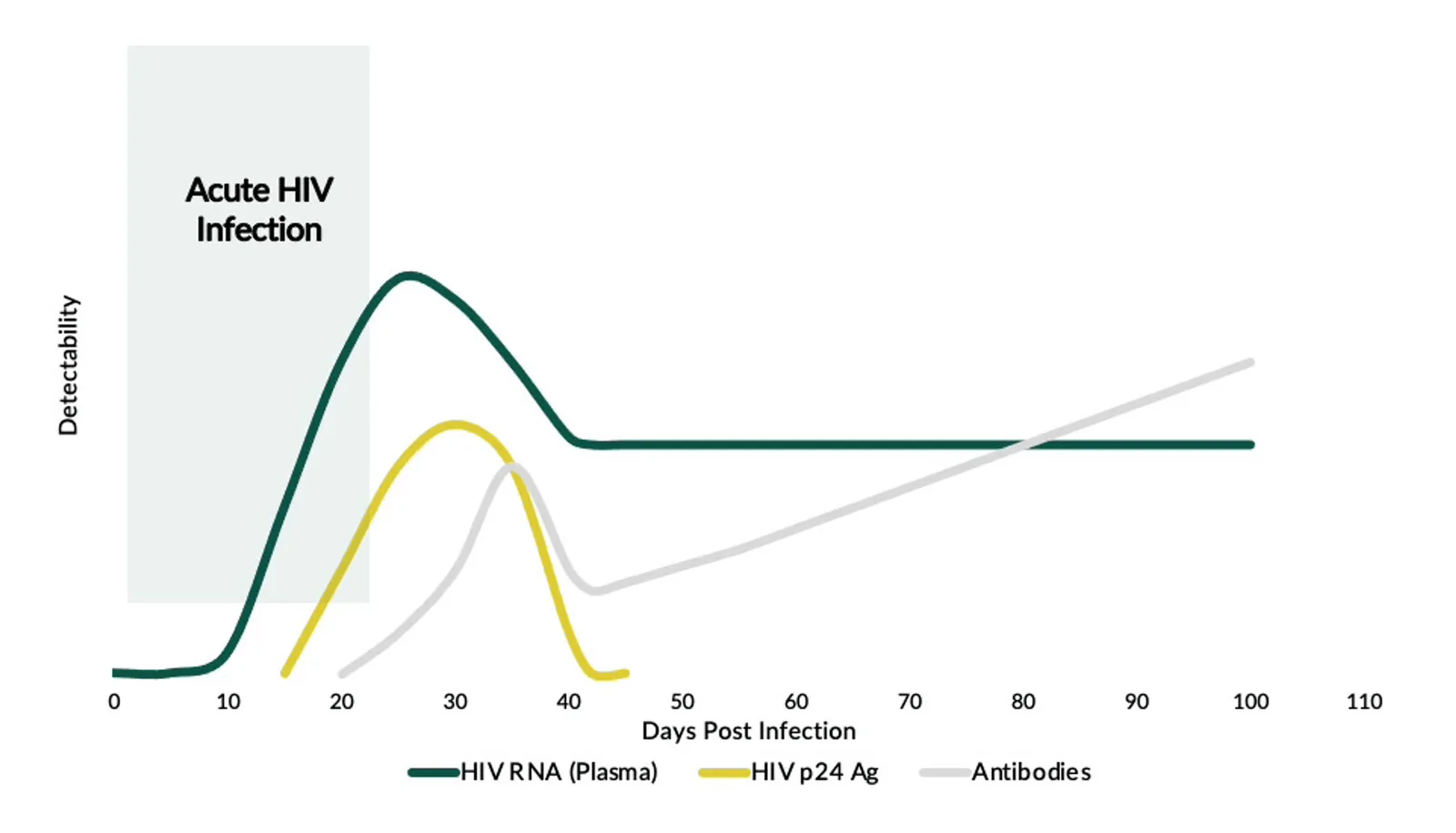
Time to positivity of HIV diagnostic markers HIV RNA is the earliest laboratory marker to become detectable following infection, followed by p24 antigen and subsequently HIV antibodies. The staggered appearance of these markers creates a diagnostic window during which the antibody-only assays may be negative despite active infection, highlighting the importance of selecting the appropriate diagnostic test based on the timing of exposure and clinical presentation. Figure created using Microsoft Excel (Microsoft® Corp., Redmond, WA). Adapted from Reference [6].

This case highlights that acute HIV does not always present in a typical, straightforward manner, and its diagnosis can be obscured by other underlying infections. In this instance, the patient’s diagnosis of streptococcal pharyngitis initially obscured the underlying HIV infection, accentuating the need for a high index of suspicion. Along with a high index of suspicion, a thorough understanding of HIV testing methods is key to a timely diagnosis. Acute HIV infection generally manifests two to four weeks after exposure to the virus and can present with non-specific symptoms, including fever, sore throat, and myalgias (Figure 1) [6]. As illustrated in Figure 1, HIV RNA becomes detectable approximately 10-14 days after infection, followed by p24 antigen approximately two to three weeks after infection, while HIV antibodies generally do not become detectable until three to six weeks after infection. Consequently, patients presenting during the early stages of infection may have negative antibody-based tests despite active HIV infection. Recognition of this diagnostic window is essential, as concurrent diagnosis, such as streptococcal pharyngitis, does not exclude HIV infection and should not preclude appropriate HIV testing when clinical suspicion remains high. 

Routine HIV testing in healthcare settings is essential to reduce missed diagnoses, particularly when behavioral risk factors are not disclosed. The CDC recommends routine HIV testing starting at age 13 in all medical settings [7]. Testing should be offered at least annually in outpatient settings, with increased frequency for high-risk patients. High-risk patients include men who have sex with men, transgender women, and risky sexual behavior deemed by any sexual behavior that increases risk of adverse outcomes such as STIs. Importantly, in the United States, a separate consent for HIV testing is not necessary in medical settings, and opportunities to screen adolescents should not be overlooked. Further, universal testing should not be limited to those who report “high-risk” behaviors, as self-reporting may be unreliable. Many patients, including the one in the case, may hesitate to disclose their sexual behaviors, further reinforcing the need for routine screening.

HIV testing considerations 

**Figure 2:**
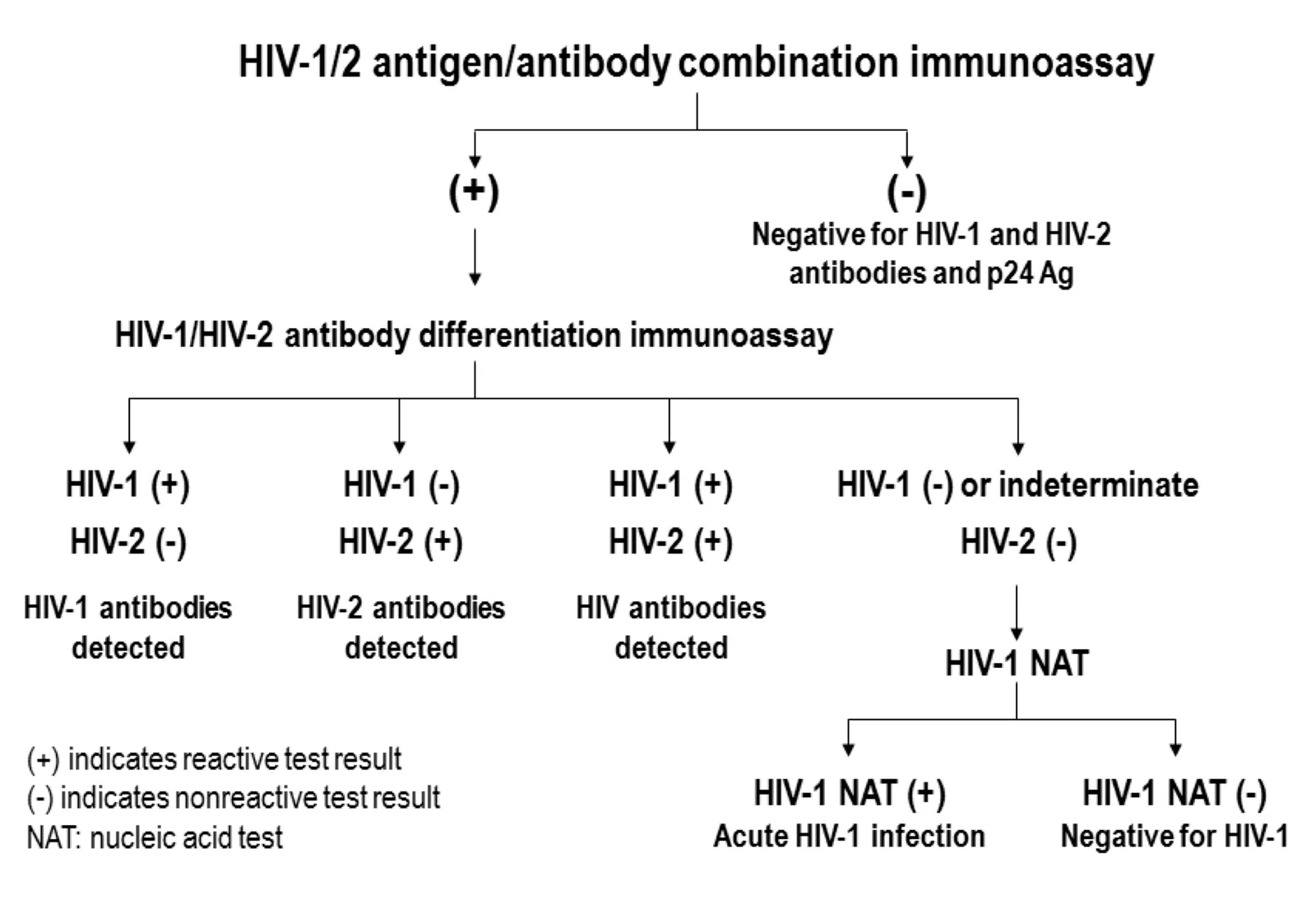
HIV diagnostic testing algorithm Recommended diagnostic algorithm for laboratory detection of HIV infection. Reactive fourth-generation HIV-1/2 antigen/antibody screening tests are followed by HIV-1/HIV-2 antibody differentiation testing, with HIV-1 NAT performed when antibody differentiation results are negative or indeterminate to distinguish acute infection from false-positive screening results. Figure adapted from Reference [6].

Not only should clinicians be versed in the importance of universal screening regardless of reported risk factors, but they should also understand the HIV diagnostic tests available and the time to positivity. As discussed, acute HIV typically presents two to four weeks after initial exposure with flu-like symptoms. The window period describes the time during which HIV tests may return negative despite an active infection. This window period occurs due to the delay between the initial infection and seroconversion or the time it takes for detectable antibodies to develop. During this period, test sensitivity is based on the test's ability to detect P24 antigens. With increasing generations of enzyme-linked immunoassays, HIV testing has been optimized from as long as 45 days to detection in first-generation tests compared to as quickly as 15 days to detection in fourth generation. Increasing target detection to include both IgM antibodies and p24 antigens in the fourth-generation immunoassay has allowed for earlier diagnosis and start of immunotherapy compared to previous-generation tests (Table 1) [8,9], making the fourth-generation test the preferred initial screening in HIV testing. Table 1 summarizes the principal HIV diagnostic assays, their targets of detection, and the approximate time after exposure at which they become positive. When testing prior to the window of fourth-generation immunoassay detection, HIV RNA viral load testing has a sensitivity cut-off of 50 copies/mL, detecting RNA in 10-15 days, with an ultrasensitive option that can detect in as soon as five days. Analytical sensitivity of this test is approximately 30,000 to 50,000 copies/mL of HIV RNA. Due to its improved detection capability, it is considered the standard initial test for HIV screening. Following a positive result with the fourth-generation HIV test, testing for HIV 1 and 2 antibody combination immunoassays, an HIV nucleic amplification test is completed. In acute HIV infection, RNA is used as a biomarker, with the NAT verifying acute HIV infection (Figure 2) [6]. Figure 2 illustrates the diagnostic algorithm for HIV testing, highlighting the role of nucleic acid testing in confirming acute HIV infection.

**Table 1:** Approximate diagnostic window periods for commonly used HIV screening and confirmatory assays HIV diagnostic tests differ in the viral or immunologic markers they detect, resulting in varying times to positivity. Nucleic acid amplification tests (HIV RNA) detect infection earliest, followed by fourth-generation antigen/antibody assays, while antibody-only assays become positive later in seroconversion. Understanding these diagnostic windows is essential for selecting the appropriate test when acute HIV infection is suspected. *Approximately 18 days post-exposure represents the earliest time at which a laboratory-based fourth-generation antigen/antibody assay can detect HIV infection in most individuals. By 45 days post-exposure, a negative result on a laboratory-based fourth-generation test is considered reliable. Abbreviations: POC, point of care; IgG, immunoglobulin G; IgM, immunoglobulin M; RNA, ribonucleic acid; and DNA, deoxyribonucleic acid References: [8,9]

Test	Target of detection	Approximate time to positivity (days)
Tests for screening		
First generation	IgG antibody	35-45
Second generation	IgG antibody	25-35
Third generation	IgM and IgG antibody	20-30
Fourth generation (lab test, blood from vein)	IgM and IgG antibody and p24 antigen	18-45*
Rapid test (POC test, finger stick)	Antigen/antibody test	18-90
Confirmatory tests		
HIV-1/HIV-2 differentiation	Antibodies to HIV-1 and/or HIV-2 antigens	23-90
Western blot	IgM and IgG antibody	35-50 (indeterminate)
		45-60 (positive)
Nucleic acid tests		
Qualitative tests	HIV RNA	10-33
	HIV DNA	10-33
Quantitative test (viral load)	HIV RNA	10-33

Testing barriers 

Potential testing barriers in routine HIV testing include stigma, fear, confidentiality concerns, and time constraints in primary care settings. Research indicates that time limitations and provider discomfort discussing sensitive topics contribute to low screening rates in adolescents [3]. Based on these barriers, programs in pediatric and adolescent medicine should be implemented to establish routine protocols to facilitate screening. Studies show that youth patients prefer provider-delivered HIV testing over client-initiated or off-site testing, highlighting the importance of a supportive and trustworthy clinical environment in promoting HIV testing uptake [10]. Universal screening of donor blood and pregnant individuals has significantly reduced HIV transmission. Applying similar universal testing protocols for adolescents should be strongly advocated for improving early detection and reducing transmission rates. 

## Conclusions

This case highlights the importance of understanding the various HIV diagnostic tests and their window periods for positivity. Clinicians must be aware of these nuances to enhance early detection and timely intervention. Maintaining a high index of suspicion for HIV in adolescent patients is crucial, as missed or delayed diagnoses can accelerate disease progression and increase transmission. Integrating HIV testing into routine adolescent care helps normalize screening and reduce stigma. Furthermore, because risk cannot always be assumed and is not always disclosed, universal testing should be the standard of care to ensure early HIV identification and treatment.
